# Supplementary figures and images for: Endothelial cell-initiated extravasation of cancer cells visualized in zebrafish
Source: PeerJ. 2014 Dec 23;2:e688. doi: 10.7717/peerj.688 (PMC4277486; doi:10.7717/peerj.688)

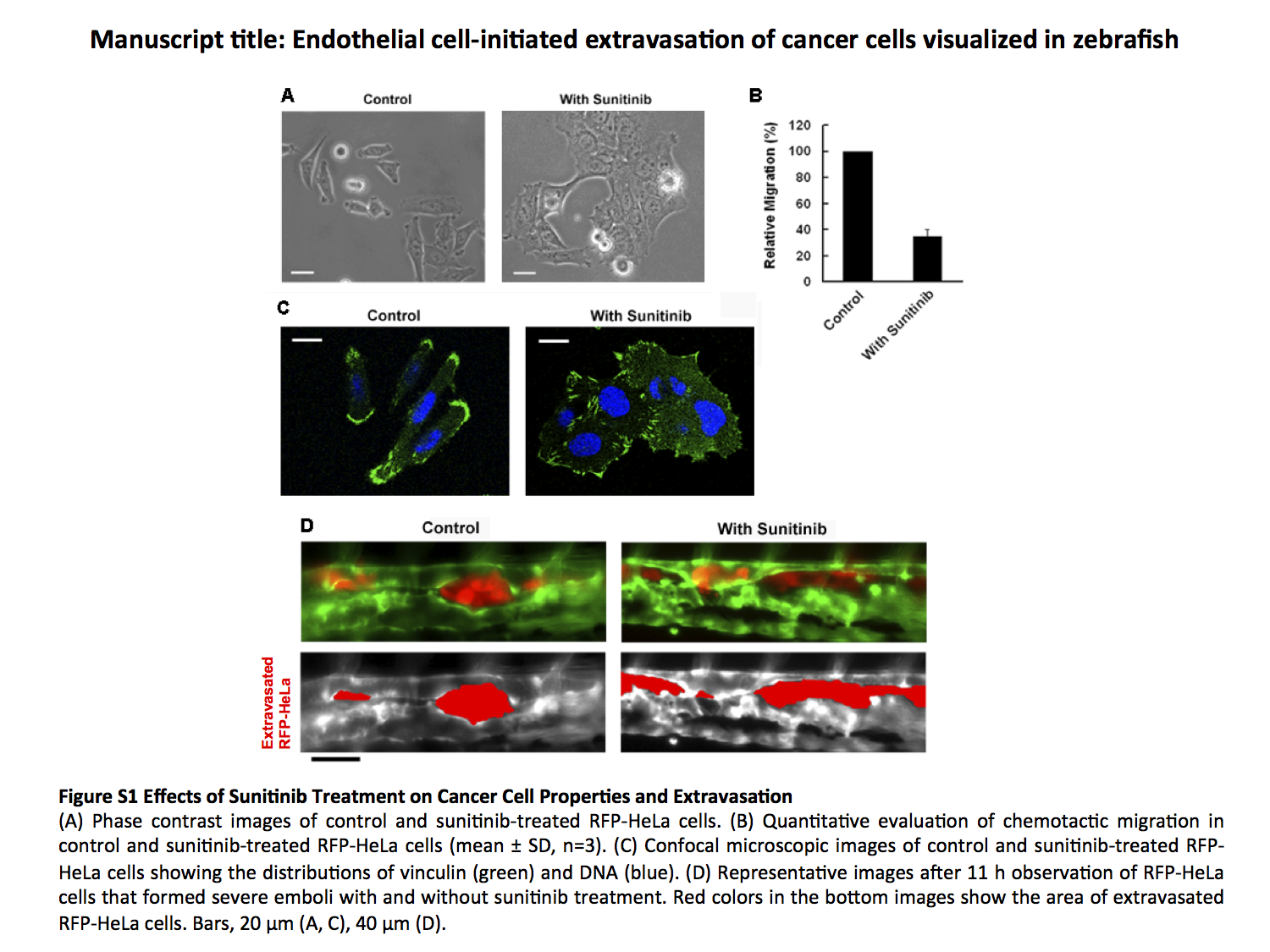

Supplement: Figure S1 — (A) Phase contrast images of control and sunitinib-treated RFP-HeLa cells. (B) Quantitative evaluation of chemotactic migration in control and sunitinib-treated RFP-HeLa cells (mean ± SD, n = 3). (C) Confocal microscopic images of control and sunitinib-treated RFP-HeLa cells showing the distributions of vinculin (green) and DNA (blue). (D) Representative images after 11 h observation of RFP-HeLa cells that formed severe emboli with and without sunitinib treatment. Red colors in the bottom images show the area of extravasated RFP-HeLa cells. Bars, 20 µm (A, C), 40 µm (D). [file peerj-02-688-s001.png]
